# Interactions between Nitrogen and Silicon in Rice and Their Effects on Resistance toward the Brown Planthopper *Nilaparvata lugens*

**DOI:** 10.3389/fpls.2017.00028

**Published:** 2017-01-23

**Authors:** Xiaoying Wu, Yaoguang Yu, Scott R. Baerson, Yuanyuan Song, Guohua Liang, Chaohui Ding, Jinbo Niu, Zhiqiang Pan, Rensen Zeng

**Affiliations:** ^1^State Key Laboratory of Conservation and Utilization of Subtropical Agro-Bioresources, College of Natural Resources and Environment, South China Agricultural University (SCAU)Guangzhou, China; ^2^Key Laboratory of Ministry of Education for Genetics, Breeding and Multiple Utilization of Crops, College of Crop Science, Fujian Agriculture and Forestry UniversityFuzhou, China; ^3^Natural Products Utilization Research Unit, United States Department of Agriculture – Agricultural Research Service, StarkvilleMS, USA

**Keywords:** nitrogen, silicon, interaction, rice, brown planthopper, growth–defense tradeoff

## Abstract

Nitrogen (N) and silicon (Si) are two important nutritional elements required for plant growth, and both impact host plant resistance toward insect herbivores. The interaction between the two elements may therefore play a significant role in determining host plant resistance. We investigated this interaction in rice (*Oryza sativa* L.) and its effect on resistance to the herbivore brown planthopper *Nilaparvata lugens* (BPH). Our results indicate that high-level (5.76 mM) N fertilization reduced Si accumulation in rice leaves, and furthermore, this decrease was likely due to decreased expression of Si transporters *OsLsi1* and *OsLsi2*. Conversely, reduced N accumulation was observed at high N fertilization levels when Si was exogenously provided, and this was associated with down-regulation of *OsAMT1;1* and *OsGS1;1*, which are involved in ammonium uptake and assimilation, respectively. Under lower N fertilization levels (0.72 and/or 1.44 mM), Si amendment resulted in increased *OsNRT1:1*, *OsGS2*, *OsFd-GOGAT*, *OsNADH-GOGAT2*, and *OsGDH2* expression. Additionally, bioassays revealed that high N fertilization level significantly decreased rice resistance to BPH, and the opposite effect was observed when Si was provided. These results provide additional insight into the antagonistic interaction between Si and N accumulation in rice, and the effects on plant growth and susceptibility to herbivores.

## Introduction

Many studies have shown that plant anti-herbivore resistance is directly linked to physiological status, thus any factors affecting a plant’s physiology could potentially alter its resistance to insect pests (Slansky, 1990; Altieri and Nicholls, 2003). Application of synthetic fertilizers alters the balance of nutrients and promotes crop growth, yet also alters the production of defense-related secondary compounds that can impact the susceptibility of a given crop to insect herbivores (Altieri and Nicholls, 2003).

The macronutrient nitrogen (N) is an essential element for growth and reproduction of both plants and animals, and has been considered critical for determining interactions between plants and their consumers, including herbivorous insects (Chen et al., 2008). Nitrogen is the most frequently used fertilizer component in crop production, and can also exert an array of bottom-up effects on herbivore populations and their natural enemies (Chen et al., 2010; Winter and Rostás, 2010).

The acquisition and effective utilization of N from host plants is key to the growth and development of most herbivorous insect species, and host plant N levels can be the most important factor affecting herbivore performance (Awmack and Leather, 2002). Numerous studies have shown that high-level N fertilizer application to crops can influence plant-insect interactions, and potentially increase growth, food consumption, survival, reproductive rates and population densities of insect herbivores (Mattson, 1980; Moon et al., 1995; Brodbeck et al., 2001; Khan and Port, 2008). Conversely, a deficiency of N may alter plant metabolism and trigger insect resistance (Comadira et al., 2015). It has been demonstrated that the increasing populations of major insect pests of rice, including planthoppers, leaffolders, and stem borers, are closely related to the long-term excessive application of N fertilizers in most of the rice growing areas in Asia (Lu et al., 2007).

Silicon (Si) is the second most abundant element in soil and accumulates to significant levels within the cells of many plant species. Although the physiological role of plant-assimilated Si has long been debated, its beneficial effects on plant resistance to both abiotic and biotic stresses, including insect herbivory, is well established (e.g., Reynolds et al., 2009; Meharg and Meharg, 2015). Two different mechanisms have been proposed to account for Si-mediated plant defense against insect herbivores. One mechanism involves amorphous Si depositing in plant tissues acting as a physical barrier, leading to increased rigidity and abrasiveness of plant tissues, thus reducing its digestibility to insect pests (Keeping and Meyer, 2006; Massey and Hartley, 2009). A second mechanism involves the ability of Si taken up from the soil solution by plant roots to induce specific plant chemical defenses and prime phytohormone-mediated defense responses via the jasmonate (JA) signaling pathway (Fawe et al., 1998; Gomes et al., 2005; Fauteux et al., 2006; Ye et al., 2013), which may include the increased release of plant volatiles which attract natural enemies of insect herbivores (Kvedaras et al., 2010).

Currently available evidence indicates that, in specific plant species, Si accumulation increases plant resistance against insect herbivores, while high N levels are associated with increased insect pest population densities. Thus, for Si-accumulating plant species, there appears to be tradeoff between Si and N accumulation with significant consequences for a plant’s ability to mount defenses against insect herbivores. In rice plants, external N application can reduce intracellular Si accumulation, which was associated by one study with a decline in Si deposition in the epidermal cell walls of leaves (Mauad et al., 2003; Tsujimoto et al., 2014). Additionally, in an earlier study working with rice seedlings it was found that Si amendment in organic soil resulted in decreased plant N contents (Deren, 1997). Although prior studies have shown that there is an interaction between N and Si uptake in plants (see also Massey et al., 2007; Lemus et al., 2008), the mechanistic basis for this interaction and its relationship to insect herbivore performance have not been directly examined.

Rice (*Oryza sativa* L.) is an important crop as well as a high Si-accumulating plant, with reported Si contents reaching levels as high as 10% of total shoot dry weight (Epstein, 1999; Ma and Yamaji, 2006). The brown planthopper (BPH), *Nilaparvata lugens* Stål (Homoptera: Delphacidae), is a phloem-feeding insect and one of the most economically deleterious insect pests of cultivated rice (Bottrell and Schoenly, 2012). It was previously shown that high N fertilization levels in rice increased survival, fecundity, and egg hatchability of BPH nymphs and adults (Lu et al., 2005), whereas silicon application enhanced rice resistance against BPH (He et al., 2015). Thus, the rice-BPH interaction represents an excellent model for further examining relationships between N uptake, Si uptake, and resistance to insect herbivores in Si-accumulating plants. In the present work, we firstly investigate the molecular mechanisms involved in the interactions between N and Si in rice plants and their effects on BPH performance, by examining the transcriptional responses of a panel of genes involved in Si and N transport and assimilation, under various fertilization regimes (two Si and three N concentrations), and in the presence and absence of BPH infestation. Our finding would provide further evidence to understand the molecular details of the interaction between Si and N in rice.

## Materials and Methods

### Plant Material and BHP Insects

Rice (*Oryza sativa* L. *cv. Shishoubaimao*), was used for this study. BPH [*Nilaparvata lugens* (Stål)] was originally obtained from rice fields on the campus of South China Agricultural University in Guangzhou, China, and maintained on rice plants in a greenhouse.

### Plant Growth and Treatment

Rice seeds were surface-sterilized with 10% (v/v) H_2_O_2_ for 10 min, rinsed with distilled water three times, then pre-imbibed in distilled water for 1 day. After pre-germination for 2 days at 28°C, seeds were transferred to culture dishes containing vermiculite and 0.5× modified Kimura B nutrient solution, and maintained in a growth chamber for 10 days. Thereafter, three plants were transplanted to a plastic box containing 1.2 L 0.5× modified Kimura B nutrient solution. After 7 additional days rice plants of uniform size were exposed to different levels of N and Si.

#### N treatment

Uniformly staged rice plants (described above) were transferred to 1× modified Kimura B nutrient solution without nitrogen. Nutrient solutions were then amended with three different N levels: (a) N-limited level: 0.72 mM; (b) basal/medium level: 1.44 mM; (c) high level, 5.76 mM. The nitrogen source used was an equimolar mixture of (NH_4_)_2_SO_4_ and Ca(NO_3_)_2_⋅4H_2_O. CaCl_2_ was also added as needed to balance the calcium levels in the different treatment solutions.

#### Si Treatment

Sodium silicate (Na_2_SiO_3_⋅9H_2_O, 1.5 mM) was also added to the above nutrient solution at different N levels. For Si-untreated plants, NaCl was also added to balance sodium levels. All treated rice plants were grown in a greenhouse with day/night temperature of 30°C/26°C, 75% relative humidity and nature daylight. The modified Kimura B nutrient solution consisted of macronutrients: 0.36 mM (NH_4_)_2_SO_4_, 0.36 mM Ca(NO_3_)_2_⋅4H_2_O, 0.27 mM K_2_SO_4_, 0.55 mM MgSO_4_⋅7H_2_O, 0.18 mM KH_2_PO_4_; and micronutrients: 20 μM EDTA-Fe, 0.77 μM ZnSO_4_⋅7H_2_O, 0.32 μM CuSO_4_⋅5H_2_O, 46.26 μM H_3_BO_3_, 9.10 μM MnCl_2_, 0.15 μM (NH_4_)_6_Mo_7_O_24_⋅4H_2_O. Nutrient solutions were replenished every 3 days during the experiment. After 30 days the rice plants grown at different N levels, with or without Si amendment, were harvested and used for follow up analyses (described below).

### Rice Dry Weight

Root and shoot systems of rice plants were harvested following exposure to the various nutrient treatment solutions (described above), dried at 70°C for 3 days, then weighed. Root/shoot ratios were then calculated from the dry weight values.

### Elemental Analysis

For measurement of N and Si contents, leaves and stems of rice plants were first harvested following exposure to the various nutrient treatment solutions (described above), dried at 70°C for 3 days, then hand-pulverized to a fine powder using a mortar and pestle. Total N contents in 2 mg dry samples were then analyzed using an Elemental Analyser (TOC select, Elementar, Germany). Total Si contents in 10 mg dry samples were then analyzed by molybdenum blue colorimetric method developed by Novozamsky et al. (1984) with modifications described by Dannon and Wydra (2004). Plant materials were dissolved in the mixture of hydrogen chloride and hydrogen fluoride (1:2), and ammonium molybdate was added into the solution as color agent. Si content in the resulting solutions was detected at 811 nm by a spectrophotometer.

### BPH Bioassays

Five emergent macropterous female BPH adults were starved for 2 h, then placed into parafilm bags which were fastened directly onto the plant shoots. Total honeydew produced by each group was then weighed following a 48 h feeding period. At the same time, the number of dead BPH were counted to determine % mortality values. Ten rice plants were used for each treatment, and the experiment was repeated twice. At 4 and 8 h after BPH infestation, the leaf sheathes were harvested for real-time PCR analysis of defense gene expression in BPH infested and un-infested rice plants.

### Quantitative Real-Time PCR Analysis

Total RNAs were extracted from 0.1 g flash-frozen, powdered leaf/root samples using the TRIzol Reagent (Life Technologies, USA) according to the manufacturer’s instructions. First-strand cDNAs were synthesized from 1 μg of total RNA using a M-MLV Reverse Transcripatase (ThermoFisher Scientific, USA) according to the manufacturer’s instructions. Real-time PCR was performed using SYBR Premix Ex Taq II (Tli RNaseH Plus, Takara, Japan) with a 7500 Fast Real-Time PCR Sequence Detection System (Applied Biosystems 7500, USA). The thermal profile used was 90 °C for 30 s, followed by 40 cycles of 95°C for 15 s, 60°C for 30 s, then 72°C for 30 s. Melting curve analysis and agarose gel electrophoresis were carried out to verify amplicon specificity. Relative transcript levels were calculated using the double-standard curves method, and the rice housekeeping gene *OsActin* was used as an endogenous control. All of the gene-specific primers used in this study are listed in **Supplementary Table S1**. All assays were performed in triplicate using three biological replicates per treatment.

### Data Analysis

The SPSS statistics 17.0 package for windows was used for statistical analysis. Data were evaluated by factorial ANOVA with treatment differences among means tested at *P* = 0.05 using a Tukey *post hoc* test.

## Results

### Effects of N and Si on Rice Biomass Accumulation

**FIGURE 1:**
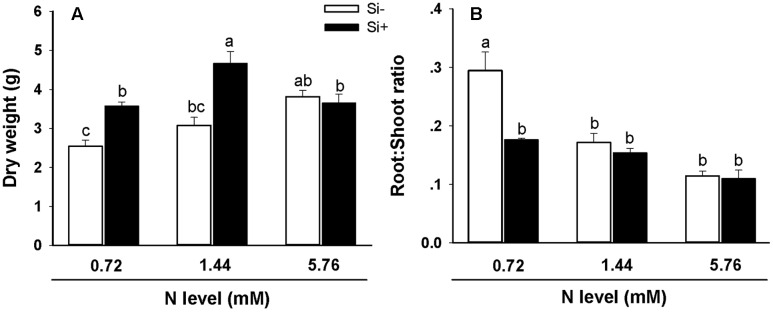
**Dry weight (A)** and root/shoot ratios **(B)** of rice plants fertilized at different nitrogen (N) levels (0.72, 1.44, and 5.76 mM) with or without silicon (Si) amendment (Si–, Si+). Values are mean ± SE (*n* = 10). Letters above bars indicate significant differences among treatments (Tukey’s multiple range test, *P* < 0.05).

In the absence of Si amendment, total dry weight values were significantly higher in plants provided with the high (5.76 mM) N treatment in comparison with plants provided with the low (0.72 mM) N treatment (3.8 g dry weight vs. 2.5 g; **Figure 1A**). For plants provided with Si in addition to either 0.72 or 1.44 mM N, dry weight values were significantly higher in comparison with plants not provided with Si but receiving the same N treatments. This stimulatory effect of Si on biomass accumulation was not observed, however, when 5.76 mM was provided (**Figure 1A**). Additionally, Si amendment led to significantly lower root/shoot ratios using the 0.72 mM N treatment relative to plants not provided with Si receiving the same N treatment (**Figure 1B**).

### Effects of N Fertilizer on Si Uptake and Translocation

**FIGURE 2:**
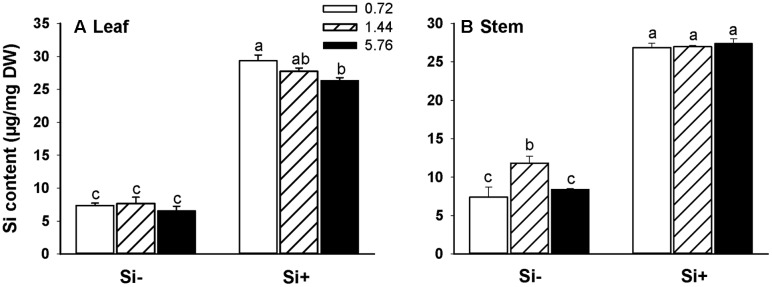
**Silicon (Si) contents of leaves** **(A)** and stems **(B)** of rice plants fertilized at different N levels (0.72, 1.44, and 5.76 mM), with or without Si amendment (Si–, Si+). Values are mean ± SE (*n* = 6). Letters above bars indicate significant differences among treatments (Tukey’s multiple range test, *P* < 0.05).

In rice plants not provided with Si, there were no significant differences observed in leaf Si accumulation levels among the different N treatments (**Figure 2A**). Si amendment resulted in dramatically higher levels of Si accumulation in both leaves and stems for all N treatments (**Figures 2A,B**). Interestingly, in plants provided with Si, Si contents were lower at the highest N treatment level (5.76 mM) than the Si contents observed in plants provided with 0.72 mM N (**Figure 2A**). In addition, in the absence of added Si, stems of 1.44 mM N-fertilized plants had higher Si contents than stems of plants provided with either 0.72 or 5.76 mM N (**Figure 2B**).

**FIGURE 3:**
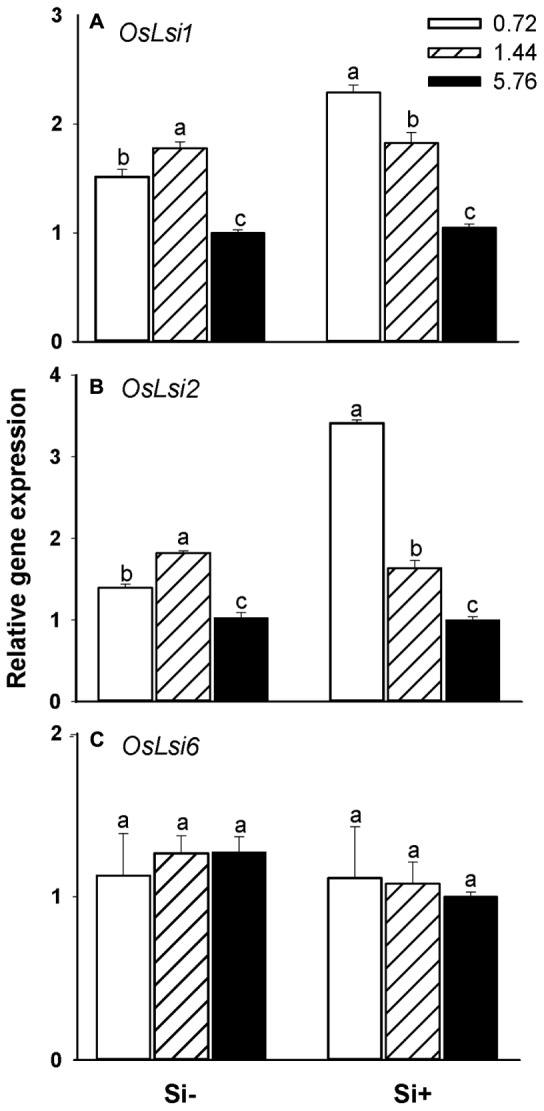
**Effects of different N fertilization levels on the relative expression of genes (A)** *OsLsi1*, **(B)** *OsLsi2*, and **(C)** *OsLsi6* involved in Si uptake and translocation in rice plants. Values are mean ± SE (*n* = 3). Letters above bars indicate significant differences among treatments (Tukey’s multiple range test, *P* < 0.05).

OsLsi1, OsLsi2, and OsLsi6 represent the major transporters involved in the uptake and translocation of Si in rice plants (Ma and Yamaji, 2008; Yamaji et al., 2008). Real-time PCR analysis revealed that in Si-treated plants the steady-state transcript levels of *OsLsi1* and *OsLsi2* decreased as higher N fertilization levels were provided (**Figures 3A,B**). Plants not provided with Si also showed decreased *OsLsi1* and *OsLsi2* transcript levels at the highest (5.76 mM) N fertilization rates in comparison with the 0.72 or 1.44 mM N treatments, however, the transcript levels for both genes were also somewhat higher in plants provided 1.44 mM N relative to plants provided with the 0.72 mM N treatment (**Figures 3A,B**). No significant changes in transcript accumulation levels were observed for *OsLsi6* regardless of the treatment solution provided (**Figure 3C**).

### Effects of Si Treatment on N Uptake and Assimilation

**FIGURE 4:**
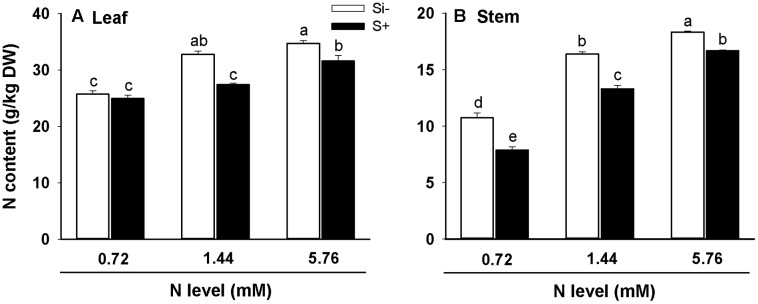
**Nitrogen contents of leaves** **(A)** and stems **(B)** of rice plants fertilized at different N levels (0.72, 1.44, and 5.76 mM), with or without Si amendment (Si–, Si+). Values are mean ± SE (*n* = 6). Letters above bars indicate significant differences among treatments (Tukey’s multiple range test, *P* < 0.05).

In both leaves and stems of rice, high (5.76 mM) levels of N resulted in significantly increased N contents relative to plants provided with low (0.72 mM) N, irrespective of Si amendment (**Figures 4A,B**). However, the addition of Si resulted in decreased N contents in both leaves and stems for nearly all treatments, with the only exception being N content levels in 0.72 mM N-treated leaves (**Figure 4A**). In leaf tissues, Si amendment lowered N contents by 16% and 8% at N treatment levels of 1.44 and 5.76 mM, respectively, and in stem tissues, Si amendment lowered N contents by 26%, 19% and 9% at N treatment levels of 0.72, 1.44, and 5.76 mM, respectively (**Figures 4A,B**).

**FIGURE 5:**
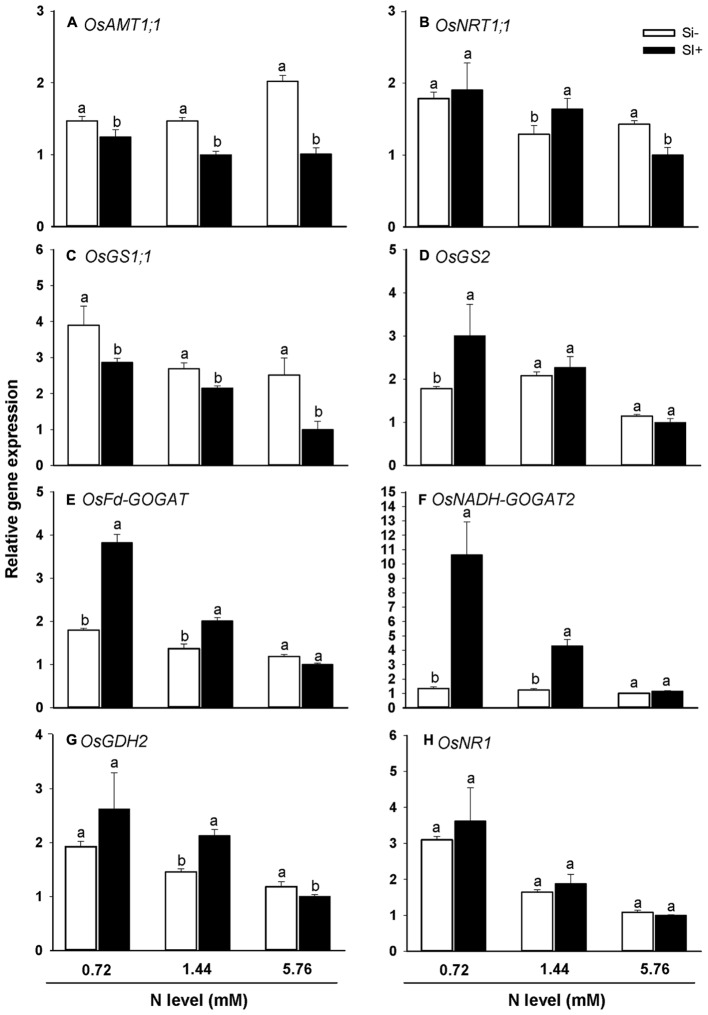
**Effects of Si amendment on the relative expression of genes involved in N uptake (A)** *OsAMT1;1* and **(B)** *OsNRT1;1* and assimilation **(C)** *OsGS1;1*, **(D)** *OsGS2*, **(E)** *OsFd-GOGAT*, **(F)** *OsNADH-GOGAT2*, **(G)** *OsGDH2*, and **(H)** *OsNR1* in rice plants. Values are mean ± SE (*n* = 3). Letters above bars indicate significant differences among treatments (Tukey’s multiple range test, *P* < 0.05).

The steady-state transcript levels of a panel of genes critical for N uptake and assimilation in rice were also monitored via qRT-PCR for the different N treatment levels in the presence and absence of Si amendment (**Figures 5A–H**). For all three N treatments, Si amendment resulted in substantially reduced transcript levels for NH_4_^+^ transporter *OsAMT1;1* (**Figure 5A**). Furthermore, a similar trend was observed for transcripts of *OsGS1;1*, which encodes the enzyme glutamine synthase 1 that uses NH_4_^+^ and glutamate to generate glutamine (**Figure 5C**). At the 0.72 mM N treatment level, Si amendment led to increased transcript levels for *OsGS2*, *OsFd-GOGAT* and *OsNADH-GOGAT2* (**Figures 5D–F**). At the 1.44 mM N treatment level, Si amendment led to increased *OsNRT1:1, OsFd-GOGAT*, *OsNADH-GOGAT2*, and *OsGDH2* transcript levels (**Figures 5B,E–G**). At the highest N treatment level (5.76 mM) Si amendment led to reduced transcript levels of *OsNRT1:1* and *OsGDH2* (**Figures 5B,G**), but had no discernible effect on transcript levels for the other four N metabolism-associated genes tested. In particular, the strong correlation observed between *OsAMT1;1* and *OsGS1;1* steady transcript levels with N accumulation levels in rice under various nutrient regimes (**Figure 4** vs. **Figures 5A,C**) strongly suggests that the observed decrease in N accumulation in plants provided with exogenous Si is due, at least in part, to decreased NH_4_^+^ uptake via *OsAMT1;1* and decreased NH_4_^+^ assimilation via *OsGS1;1*.

### Effects of N Levels and Si Amendment on Rice Resistance to BPH

**FIGURE 6:**
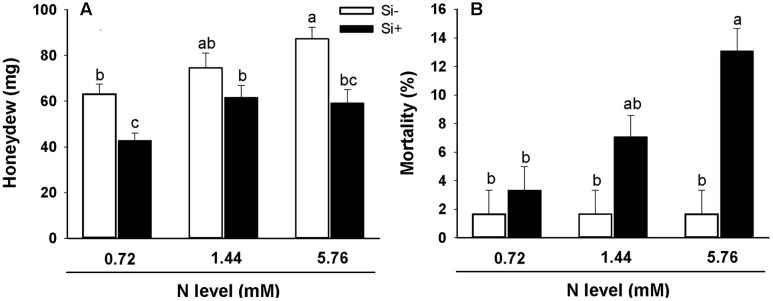
**Honeydew production (A)** and percent mortality **(B)** of BPH feeding on rice plants fertilized at different N levels (0.72, 1.44, and 5.76 mM), with or without Si amendment (Si–, Si+). Values are mean ± SE (*n* = 20). Letters above bars indicate significant differences among treatments (Tukey’s multiple range test, *P* < 0.05).

Bioassays showed significantly increased BPH honeydew excretion (an indicator of food intake) for insects feeding on rice plants grown using the high (5.76 mM) N treatment relative to insects feeding on plants provided with low (0.72 mM) N without Si amendment (**Figure 6A**). However, when Si was also provided, honeydew excretion was reduced in comparison with insects feeding on plants not provided with Si receiving the same N treatment. Interestingly, BPH mortality was unaffected by host plant N treatment regime (0.72 vs. 1.44 vs. 5.76 mM N) on Si-untreated plants (**Figure 6B**). However, BPH feeding on plants fertilized with the high (5.76 mM) N treatment solution amended with Si exhibited significantly higher percent mortality than insects feeding on plants not provided with Si (**Figure 6B**).

**FIGURE 7:**
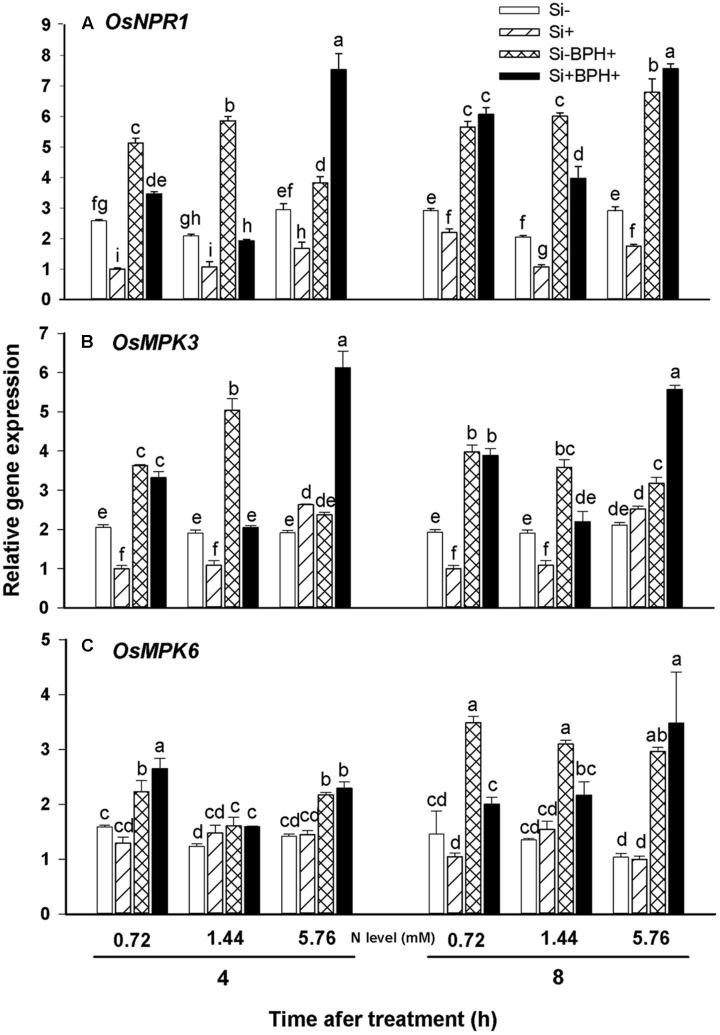
**Relative expression of OsNPR1 (A)**, *OsMPK3* **(B)**, and *OsMPK6* **(C)** in rice plants infested with or not with BPH. Values are mean ± SE (*n* = 3). Letters above bars indicate significant differences among treatments (Tukey’s multiple range test, *P* < 0.05).

Non-expressor of PR genes1 (NPR1) is the central regulator in SA-mediated plant anti-herbivore defense (Wasternack and Hause, 2013). Real-time PCR analysis showed that Si amendment reduced *OsNPR1* transcript levels in plants relative to levels observed in Si-untreated plants for all N fertilization levels tested without BPH (**Figure 7A**). BPH infestation led to significantly increased *OsNPR1* transcript levels in both Si-treated and untreated plants at 4 and 8 h after BPH inoculation. Although transcript levels of *OsNPR1* were not higher in Si-treated relative to Si-untreated plants at the two lower N fertilization levels (0.72 and 1.44 mM) at 4 and 8 h after BPH feeding, the magnitude of induction following BPH infestation was generally greater in Si-treated (by 2.76 and 3.73-fold for 0.72 and 1.44 mM N level, respectively) versus untreated plants (by 1.94 and 2.94-fold for 0.72 and 1.44 mM N level, respectively) at 8 h. Furthermore, in high N-fertilized (5.76 mM) plants, Si treatment significantly increased *OsNPR1* transcript levels in response to BPH infestation after 4 or 8 h compared to Si-untreated plants, in both the steady-state transcript levels observed as well as the magnitude of induction (**Figure 7A**).

Mitogen-activated protein kinase (MAPK) cascades also play an important role in plant signaling pathways involved in anti-herbivore defense responses (Pitzschke et al., 2009). Real-time PCR analyses revealed that Si treatment led to decreased *OsMPK3* transcript levels in low N-fertilized rice, but tended to increase the transcripts under high N fertilization (**Figure 7B**). Overall, Si-amendment did not appear to significantly affect *OsMPK6* expression (**Figure 7C**). At 8 h post-BPH infestation, *OsMPK3* and *OsMPK6* transcript levels in both Si-treated and untreated plants were significantly induced by BPH infestation relative to non-BPH infested plants. In Si-untreated plants, *OsMPK3* transcript levels were much higher under low N fertilization (0.72 and 1.44 mM) than transcript levels observed under high N fertilization (5.76 mM) at 4 and 8 h post-BPH inoculation (**Figure 7B**), but this was not the case for *OsMPK6* (**Figure 7C**). As was also the case for *OsNPR1* transcript levels observed in plants receiving high N fertilization, Si treatment significantly increased *OsMPK3* transcript levels, as well as the magnitude of induction observed relative to Si-untreated plants at 4 and 8 h post-BPH infestation (**Figure 7B**). In low N fertilization, the fold-change in *OsMPK3* transcript levels in response to BPH infestation was also increased in Si-treated versus untreated plants after BPH inoculation at 8 h.

## Discussion

Both nitrogen and silicon play an important role in rice resistance toward herbivorous insects. The application of fertilizer causes changes in plant nutrient status, which may in turn impact plant resistance levels against herbivores (De Kraker et al., 2000). In the present study, we found that high N fertilization levels significantly reduced the Si content in rice leaves (**Figure 2A**), consistent with observations made in previous studies (Massey et al., 2007; Mauad et al., 2013; Tsujimoto et al., 2014). To examine the molecular basis for this effect, we first monitored steady-state transcript levels of *OsLsi1*, *OsLsi2*, and *OsLsi6* which are directly involved in Si uptake and translocation in rice, by qRT-PCR analysis. OsLsi1 and 2 are responsible for Si uptake in roots, and OsLsi6 is involved in the distribution of Si within shoot tissues. Both OsLsi1 and OsLsi6 belong to the nodulin-26 intrinsic protein III (NIP III) subgroup of aquaporins, while OsLsi2 is a secondary active anion transporter (Ma and Yamaji, 2008; Yamaji et al., 2008). Importantly, we found that high N fertilization levels led to decreased *OsLsi1* and *OsLsi2* transcript levels (**Figures 3A,B**), but did not influence *OsLsi6* expression (**Figure 3C**). The observed reduced accumulation of Si in leaves of rice provided with high levels of N (**Figure 2A**) is therefore likely attributable to decreased OsLsi1 and/or OsLsi2 activity under these conditions. In leaves of Si-untreated plants where Si contents were much lower, higher N fertilization levels did not significantly affect leaf Si contents (**Figure 2A**) even though *OsLsi1* and *OsLsi2* steady-state transcript levels were reduced by 34 and 26%, respectively (**Figures 3A,B**) in plants provided with 5.76 mM N relative to plants provided with 0.72 mM N. Additionally, *OsLsi1* and *OsLsi2* transcript levels were significantly higher in Si-untreated plants fertilized with 1.44 mM N, compared with plants provided with 0.72 mM N (**Figures 3A,B**), and this corresponded with significantly higher stem Si contents under these nutrient conditions (**Figure 2B**). Differences in stem Si contents among plants provided with Si at various N levels were not observed. Taken together, the experimental results shown in **Figures 2** and **3** suggest that increased N fertilization negatively impacts Si accumulation in rice plants via reduced *OsLsi1* and *OsLsi2* expression, and available Si levels as well as tissue-specific factors also influence these interactions.

The results obtained in the present work also provide further evidence for an antagonistic interaction occurring between Si and N uptake in rice, as Si-amendment to the treatment solutions significantly reduced N contents in both leaves and stems, irrespective of the N levels provided (**Figures 4A,B**). The only exception to this was leaf tissues fertilized with limited (0.72 mM) N, thus available N levels are also likely to play a role in this interaction. These results are in agreement with prior studies suggesting interference of plant N uptake by Si (e.g., Deren, 1997; Massey et al., 2007).

In addition to observed inhibitory effects of increasing N on the expression of genes required for Si uptake (**Figures 3A,B**), the present work also showed that exogenously-provided Si can in turn inhibit the expression of genes important for N uptake and assimilation in rice, thus providing some insight into the mechanism underlying this interaction (**Figure 5**). OsAMT1;1 and OsNRT1;1 are two of the major transporters involved in NH_4_^+^ and NO_3_^-^ uptake in rice, respectively, and glutamine synthetase (GS), glutamate synthase (Fd-GOGAT and NADH-GOGAT), glutamate dehydrogenase (GDH) and NO_3_^-^ reductase (NR) are responsible for NH_4_^+^ or NO_3_^-^ assimilation in plant N metabolism (Tabuchi et al., 2007; Rennenberg et al., 2010). Our results suggest that *OsAMT1;1* and *OsGS1;1* may play an important role in Si-mediated inhibition of N uptake and assimilation, since transcript levels for both genes were significantly reduced in Si-treated plants at all three N levels relative to Si-untreated plants (**Figures 5A,C**). Interestingly, at lower (0.72 and 1.44 mM) N fertilization levels, Si treatment tended to correlate with elevated *OsNRT1;1*, *OsGS2*, *OsFd-GOGAT*, *OsNADH-GOGAT2* and *OsGDH2* transcript levels (**Figures 5B,D–G**), potentially identifying a feedback response mechanism operating under reduced N availability, which could also at least partially account for the observed increases in plant dry weight and decreases in root/shoot ratios under reduced N conditions (**Figures 1A,B**; see also Ericsson, 1995). In addition, fertilization at the highest N levels led to decreased *OsNRT1;1* and *OsGDH2* transcript levels (**Figures 5B,G**) which would presumably further reduce rates of N uptake and assimilation. Thus, the results further suggest that in rice Si amendment influences N uptake and assimilation rates differentially under conditions of low versus high N. The present study as well as prior work clearly show that there is an interaction between N and Si accumulation in rice plants, which in most cases appears to be mutually antagonistic. While we have provided, preliminary data describing some molecular details of these interactions, much more work will be required to fully elucidate the regulatory mechanisms involved.

Nutrient status not only influences plant growth rates, but also effects the defensive capabilities of plants (Ishiguro, 2001; Altieri and Nicholls, 2003), therefore interactions between N and Si in rice may have important consequences for plant-insect herbivore interactions. High N fertilization levels increased the honeydew excretion of BPH (**Figure 6A**), suggesting that high N input may increase the risk of BPH infestation in rice plants. Various inhibitory effects of Si on insect herbivore infestations have been documented by prior studies, including reports of increased survival rates and increased stalk damage with stalk borer infestations in sugarcane grown under elevated N levels, while Si amendment reduced stalk damage (Keeping et al., 2014). In our work, Si treatment effectively reduced the BPH honeydew excretion in the rice plants, and additionally in high N-fertilized rice, the mortality of BPH feeding on Si-treated plants was significantly increased relative to Si-untreated plants (**Figures 6A,B**).

An increased physical barrier produced by Si deposition beneath leaf cuticles has long been considered as a major mechanism underlying Si-mediated plant resistance to insect pests (Reynolds et al., 2009). Silicon deposition, occurring mainly as amorphous silica in the form of phytoliths in the epidermis (Currie and Perry, 2007), increases the rigidity and abrasiveness of plant tissues, thereby creating a mechanical barrier and reducing their digestibility to insect herbivores (Goussain et al., 2005; Kvedaras et al., 2007; Massey and Hartley, 2009). Recent studies have demonstrated that Si-mediated anti-herbivore defense is inducible and chemically mediated (Gomes et al., 2005; Kvedaras et al., 2010; Ye et al., 2013).

It is well-known that the salicylic acid (SA) and jasmonic acid (JA) signaling pathways play vital roles in plant chemical defense responses, and phloem-feeding insects like BPH tend to induce SA-mediated plant anti-herbivore defense, and *NPR1* represents the major regulatory gene involved in SA signaling pathway (Moran and Thompson, 2001; Wasternack and Hause, 2013). MAPK cascades are involved in the transduction of various stimuli, including abiotic and biotic stressors, and MPK3 and MPK6 act as positive mediators of defense responses in plants (Takahashi et al., 2007; Pitzschke et al., 2009). In the present work, we also found that BPH infestation significantly increased the expression of *OsNPR1*, *OsMPK3*, and *OsMPK6* in both Si-treated and untreated plants following BPH inoculation (**Figure 7**). Our previous work showed that SA levels in BPH-infested rice were significantly higher than those in the non-infested plants (Ye et al., 2012), suggesting that SA-mediated defense responses can be induced by BPH in rice. More work should be conducted to confirm the detailed mechanisms involved. In Si-untreated plants, *OsNPR1* and *OsMPK3* transcript levels were much higher under low N fertilization (0.72 and 1.44 mM) than transcript levels observed under high N fertilization (5.76 mM) at 4 h post-BPH inoculation, indicating that rice plants may responded more rapidly to BPH infestation under lower N in by up-regulation of *OsNPR1* and *OsMPK3* genes. The different expression pattern between *OsMPK3* and *OsMPK6* in response to BPH infestation (**Figures 7B,C**) indicates that the two *MAPKS* may be mediated independently by different signaling pathways in rice response to BPH, and defense strategies are likely to be quite complicated. At high N fertilization level, Si amendment significantly increased *NPR1* and *MPK3* transcript level relative to Si-untreated plants after BPH inoculation at 4 and 8 h (**Figures 7A,B**), suggesting that in addition to physical barrier, Si-mediated rice resistance to BPH is also inducible and much possibly SA-mediated.

## Conclusion

Our results show that an interaction exists between N and Si in rice. High N fertilization levels lead to reduced Si accumulation, due likely to decreased expression of *OsLsi1* and *OsLsi2*, which are the major transporters responsible for Si uptake and transport in rice. Our results also suggest that *OsAMT1;1* and *OsGS1;1* may play an important role in Si-mediated inhibition of N accumulation in rice, since transcript levels for both genes were significantly reduced in Si-treated plants relative to Si-untreated plants at all N fertilization levels tested. At lower N fertilization levels, Si treatment tended to increase the transcript levels of certain genes involved in N uptake and assimilation, potentially identifying a feedback response mechanism operating under reduced N availability. Si amendment also enhanced rice resistance to BPH, thus the strong interaction between Si and N accumulation in rice may have important implications for the resistance of rice plants to insect herbivores when grown in the presence of high N or Si-amended soils.

## Author Contributions

XW and RZ designed the research; XW, YY, GL, YS, CD, and JN performed the research; XW, YS, SB, ZP, and RZ analyzed data; XW, SB, ZP, and RZ wrote the manuscript.

## Conflict of Interest Statement

The authors declare that the research was conducted in the absence of any commercial or financial relationships that could be construed as a potential conflict of interest.

## References

[B1] Altieri M. A. Nicholls C. I. (2003). Soil fertility management and insect pests: harmonizing soil and plant health in agroecosystems. *Soil. Till. Res.* 72 203–211. 10.1016/S0167-1987(03)00089-8

[B2] Awmack C. S. Leather S. R. (2002). Host plant quality and fecundity in herbivorous insects. *Annu. Rev. Entomol.* 47 817–844. 10.1146/annurev.ento.47.091201.145300 11729092

[B3] Bottrell D. G. Schoenly K. G. (2012). Resurrecting the ghost of green revolutions past: the brown planthopper as a recurring threat to high-yielding rice production in tropical Asia. *J. Asia Pac. Entomol.* 15 122–140. 10.1016/j.aspen.2011.09.004

[B4] Brodbeck B. Stavisky J. Funderburk J. Andersen P. Olson S. (2001). Flower nitrogen status and populations of *Frankliniella occidentalis* feeding on *Lycopersicon esculentum*. *Entomol. Exp. Appl.* 99 165–172. 10.1673/031.011.0141

[B5] Chen Y. Olson D. M. Ruberson J. R. (2010). Effects of nitrogen fertilization on tritrophic interactions. *Arthropod Plant Inte.* 4 81–94. 10.1111/1744-7917.12123

[B6] Chen Y. Ruberson J. R. Olson D. M. (2008). Nitrogen fertilization rate affects feeding, larval performance, and oviposition preference of the beet armyworm, *Spodoptera exigua*, on cotton. *Entomol. Exp. Appl.* 126 244–255. 10.1111/j.1570-7458.2007.00662.x

[B7] Comadira G. Rasool B. Karpinska B. Morris J. A. Verrall S. R. Hedley P. E. (2015). Nitrogen deficiency in barley (*Hordeum vulgare*) seedlings induces molecular and metabolic adjustments that trigger aphid resistance. *J. Exp. Bot.* 66 3639–3655. 10.1093/jxb/erv276 26038307 PMC4463806

[B8] Currie H. A. Perry C. C. (2007). Silica in plants: biological, biochemical and chemical studies. *Ann. Bot.* 100 1383–1389. 10.1093/aob/mcm247 17921489 PMC2759229

[B9] Dannon E. A. Wydra K. (2004). Interaction between silicon amendment, bacterial wilt development and phenotype of *Ralstonia solanacearum* in tomato genotypes. *Physiol. Mol. Plant Pathol.* 64 233–243. 10.1016/j.pmpp.2004.09.006

[B10] De Kraker J. Rabbinge R. Huis A. V. van Lenteren J. C. Heong K. L. (2000). Impact of nitrogenous-fertilization on the population dynamics and natural control of rice leaf folder (Lep.: Pyralidae). *Int. J. Pest. Manag.* 46 225–235. 10.1080/096708700415571

[B11] Deren C. W. (1997). Changes in nitrogen and phosphorus concentrations on silicon-fertilized rice grown on organic soil. *J. Plant Nutr.* 20 765–771. 10.1080/01904169709365292

[B12] Epstein E. (1999). Silicon. *Annu. Rev. Plant Physiol. Plant. Mol. Biol.* 50 641–664. 10.1146/annurev.arplant.50.1.641 15012222

[B13] Ericsson T. (1995). Growth and shoot: root ratio of seedlings in relation to nutrient availability. *Plant Soil.* 168 205–214. 10.1007/BF00029330

[B14] Fauteux F. Chain F. Belzile F. Menzies J. G. Bélanger R. R. (2006). The protective role of silicon in the *Arabidopsis*-powdery mildew pathosystem. *Proc. Natl. Acad. Sci. U.S.A.* 103 17554–17559. 10.1073/pnas.0606330103 17082308 PMC1859967

[B15] Fawe A. Abou-Zaid M. Menzies J. G. Bélanger R. R. (1998). Silicon-mediated accumulation flavonoid phytoalexins cucumber. *Phytopathology* 88 396–401. 10.1094/PHYTO.1998.88.5.396 18944917

[B16] Gomes F. B. Moraes J. C. Santos C. D. Goussain M. M. (2005). Resistance induction in wheat plants by silicon and aphids. *Sci. Agric.* 62 547–551. 10.1603/EN13234

[B17] Goussain M. M. Prado E. Moraes J. C. (2005). Effect of silicon applied to wheat plants on the biology and probing behaviour of the greenbug *Schizaphis graminum* (Rond.) (Hemiptera: Aphidiae). *Neotrop. Entomol.* 34 807–813. 10.1590/S1519-566X2005000500013

[B18] He W. Q. Yang M. Li Z. H. Qiu J. L. Liu F. Qu X. S. (2015). High levels of silicon provided as nutrient in hydroponic culture enhances rice plant resistance to brown planthopper. *Crop Prot.* 67 20–25. 10.1016/j.cropro.2014.09.013

[B19] Ishiguro K. (2001). Improving management strategy for rice blast disease using a simulation model of rice leaf blast epidemics. *Bull. Tohoku. Natl. Agric. Exp. Stn.* 99 1–110.

[B20] Keeping M. G. Meyer J. H. (2006). Silicon-mediated resistance of sugarcane to *Eldana saccharina* Walker (Lepidoptera: Pyralidae): effects of silicon source and cultivar. *J. Appl. Entomol.* 130 410–420. 10.1111/j.1439-0418.2006.01081.x

[B21] Keeping M. G. Miles N. Sewpersad C. (2014). Silicon reduces impact of plant nitrogen in promoting stalk borer (*Eldana saccharina*) but not sugarcane thrips (*Fulmekiola serrata*) infestations in sugarcane. *Front. Plant Sci.* 5:289 10.3389/fpls.2014.00289 PMC4064666 24999349

[B22] Khan M. Port G. (2008). Performance of clones and morphs of two cereal aphids on wheat plants with high and low nitrogen content. *Entomol. Sci.* 11 159–165. 10.1111/j.1479-8298.2008.00262.x

[B23] Kvedaras O. L. An M. Choi Y. S. Gurr G. M. (2010). Silicon enhances natural enemy attraction and biological control through induced plant defences. *Bull. Entomol. Res.* 100 367–371. 10.1017/S0007485309990265 19737442

[B24] Kvedaras O. L. Keeping M. G. Goebel F. R. Byrne M. J. (2007). Larval performance of the pyralid borer *Eldana saccharina* walker and stalk damage in sugarcane: influence of plant silicon, cultivar and feeding site. *Int. J. Pest. Manag.* 53 183–194. 10.1080/09670870601110956

[B25] Lemus R. Brummer E. C. Burras C. L. Moore K. J. Barker M. F. Molstad N. E. (2008). Effects of nitrogen fertilization on biomass yield and quality in large fields of established switchgrass in southern Iowa, USA. *Biom. Bioenergy* 32 1187–1194. 10.1016/j.biombioe.2008.02.016

[B26] Lu Z. X. Heong K. L. Yu X. P. Hu C. (2005). Effects of nitrogen on the tolerance of brown planthopper, *Nilaparvata lugens*, to adverse environmental factors. *Insect Sci.* 12 121–128. 10.1111/j.1744-7917.2005.00014.x

[B27] Lu Z. X. Yu X. P. Heong K. L. Hu C. (2007). Effect of nitrogen fertilizer on herbivores and its stimulation to major insect pests in rice. *Rice Sci.* 14 56–66. 10.1016/S1672-6308(07)60009-2

[B28] Ma J. F. Yamaji N. (2006). Silicon uptake and accumulation in higher plants. *Trends Plant Sci.* 11 392–397. 10.1016/j.tplants.2006.06.007 16839801

[B29] Ma J. F. Yamaji N. (2008). Functions and transport of silicon in plants. *Cell Mol. Life Sci.* 65 3049–3057. 10.1007/s00018-008-7580-x 18560761 PMC11131740

[B30] Massey F. P. Ennos A. R. Hartley S. E. (2007). Herbivore specific induction of silica-based plant defences. *Oecologia* 152 677–683. 10.1007/s00442-007-0703-5 17375331

[B31] Massey F. P. Hartley S. E. (2009). Physical defences wear you down: progressive and irreversible impacts of silica on insect herbivores. *J. Anim. Ecol.* 78 281–291. 10.1111/j.1365-2656.2008.01472.x 18771503

[B32] Mattson W. J. Jr. (1980). Herbivory in relation to plant nitrogen content. *Annu. Rev. Ecol. Syst.* 11 119–161. 10.1146/annurev.es.11.110180.001003

[B33] Mauad M. Crusciol C. A. C. Grassi Filho H. Rodrigues Machado S. (2013). Silica deposition and rate the nitrogen is silicon in rice. *Semin Cienc. Agrar.* 34 1653–1661.

[B34] Mauad M. Grassi H. Crusciol C. A. C. (2003). Silicon contents in soil and in highland rice plants under different doses of silicon and nitrogen fertilization. *Rev. Bras. Cienc. Solo.* 27 867–873. 10.1590/S0100-06832003000500011

[B35] Meharg C. Meharg A. A. (2015). Silicon, the silver bullet for mitigating biotic and abiotic stress, and improving grain quality, in rice? *Environ. Exp. Bot.* 120 8–17. 10.1016/j.envexpbot.2015.07.001

[B36] Moon C. E. Lewis B. E. Murray L. Sanderson S. M. (1995). Russian wheat aphid (Homoptera: Aphididae) development, reproduction, and longevity on hydroponically grown wheat with varying nitrogen levels. *Environ. Entomol.* 24 367–371. 10.1093/ee/24.2.367

[B37] Moran P. J. Thompson G. A. (2001). Molecular response to aphid feeding in *Arabidopsis* in relation to plant defense pathways. *Plant. Physiol.* 125 1074–1085. 10.1104/pp.125.2.1074 11161062 PMC64906

[B38] Novozamsky I. Van Eck R. Houba V. J. G. (1984). A rapid determination of silicon in plant material. *Commun. Soil Sci. Plant Anal.* 15 205–211. 10.1080/00103628409367470

[B39] Pitzschke A. Schikora A. Hirt H. (2009). MAPK cascade signaling networks in plant defence. *Curr. Opin. Plant Biol.* 12 1–6. 10.1016/j.pbi.2009.06.008 19608449

[B40] Rennenberg H. Wildhagen H. Ehlting B. (2010). Nitrogen nutrition of poplar trees. *Plant Biol.* 12 275–291. 10.1111/j.1438-8677.2009.00309.x 20398235

[B41] Reynolds O. L. Keeping M. G. Meyer J. H. (2009). Silicon-augmented resistance of plants to herbivorous insects: a review. *Ann. Appl. Biol.* 155 171–186. 10.1111/j.1744-7348.2009.00348.x

[B42] Slansky F. (1990). Insect nutritional ecology as a basis for studying host plant resistance. *Florida Entomol.* 73 354–378. 10.2307/3495455

[B43] Tabuchi M. Abiko T. Yamaya T. (2007). Assimilation of ammonium ions and reutilization of nitrogen in rice (*Oryza sativa* L.). *J. Exp. Bot.* 58 2319–2327. 10.1093/jxb/erm016 17350935

[B44] Takahashi F. Yoshida R. Ichimura K. Mizoguchi T. Seo S. Yonezawa M. (2007). The mitogen-activated protein kinase cascade MKK3-MPK6 is an important part of jasmonate signal transduction pathway in *Arabidopsis*. *Plant Cell* 19 805–818. 10.1105/tpc.106.046581 17369371 PMC1867372

[B45] Tsujimoto Y. Muranaka S. Saito K. Asai H. (2014). Limited Si-nutrient status of rice plants in relation to plant-available Si of soil, nitrogen fertilizer application, and rice-growing environments across Sub-Saharan Africa. *Filed Crop Res.* 155 1–9. 10.1016/j.fcr.2013.10.003

[B46] Wasternack C. Hause B. (2013). Jasmonates: biosynthesis, perception, signal transduction and action in plant stress response, growth and development. An update to the 2007 review in annals of botany. *Ann. Bot.* 111 1021–1058. 10.1093/aob/mct067 23558912 PMC3662512

[B47] Winter T. R. Rostás M. (2010). Nitrogen deficiency affects bottom-up cascade without disrupting indirect plant defense. *J. Chem. Ecol.* 36 642–651. 10.1007/s10886-010-9797-z 20443049

[B48] Yamaji N. Mitatni N. Ma J. F. (2008). A transporter regulating silicon distribution in rice shoots. *Plant Cell* 20 1381–1389. 10.1105/tpc.108.059311 18515498 PMC2438455

[B49] Ye M. Luo S. M. Xie J. F. Li Y. F. Xu T. Liu Y. (2012). Silencing COI1 in rice increases susceptibility to chewing insects and impairs inducible defense. *PLoS ONE* 4:e36214 10.1371/journal.pone.0036214 PMC3338713 22558386

[B50] Ye M. Song Y. Y. Long J. Wang R. L. Baerson S. R. Pan Z. Q. (2013). Priming of jasmonate-mediated antiherbivore defense responses in rice by silicon. *Proc. Natl. Acad. Sci. U.S.A.* 110 3631–3639. 10.1073/pnas.1305848110 PMC3780902 24003150

