# Supplementary material for: Interactions between Nitrogen and Silicon in Rice and Their Effects on Resistance toward the Brown Planthopper Nilaparvata lugens
Source: Front Plant Sci. 2017 Jan 23;8:28. doi: 10.3389/fpls.2017.00028 (PMC5253352; doi:10.3389/fpls.2017.00028)
Supplement: Supplementary file 1 [file Table_1.DOC]

**Supporting information**

**Table S1** Gene-speciﬁc primers for quantitative RT-PCR

| **Gene name** | **Forward primer (5’-3’)** | **Reverse primer (5’-3’)** | **Reference** |
| --- | --- | --- | --- |
| *OsActin* | TGGACAGGTTATCACCATTGGT | CCGCAGCTTCCATTCCTATG | Ye et al., 2013 |
| *OsAMT1;1* | GCCTCCAACAGCAACAACC | CCAAACAGAAACTGGCAATCA | Li et al., 2009 |
| *OsNRT1;1* | GGGCAGAGTTCAGCAATCG | GGAAGGACGCCGCAGGT | Lin et al., 2000 |
| *OsGS1;1* | CCGTCTGTCGGCATTTCTG | GGGATGGGCTTGGGGTC | Kusano et al., 2010 |
| *OsGS2* | AGTATGCGTGAAGATGGAGGAT | GCCCCACCCGAATAGAGC | Kusano et al., 2010 |
| *OsFd-GOGAT* | TGGTCTCCGCCCAGCAC | CAGTTTGTAGGTCAACCGTTATCAT | Kusano et al., 2010 |
| *OsNADH-GOGAT2* | CCTGTCGAAGGATCGTGAAGGTCAAACC | TGCATGGCCCTACTGTCTTCGCATCA | Kusano et al., 2010 |
| *OsGDH1* | TTCTTCCTTCCCACTACCAAAC | TCCCAAGCAGCGAGCC | Kusano et al., 2010 |
| *OsNR1* | CCTACTACTAAATTATACGCACCG | CAGGAAGGAATCAACCGCTA | Choi and Kleinhofs, 1989 |
| *OsLsi1* | CGGTGGATGTGATCGGAACCA | CGTCGAACTTGTTGCTCGCCA | Ye et al., 2013 |
| *OsLsi2* | ATCTGGGACTTCATGGCCC | ACGTTTGATGCGAGGTGG | Ye et al., 2013 |
| *OsLsi6* | GAGTTCGACAACGTCTAATCGC | AGTACACGGTACATGTATACACG | Ye et al., 2013 |
| *OsNPR1* | TGCTGCTCACTGAAGGACAGAC | ATCTGCAAGTGCGAGATCCAA | Li et al., 2013 |
| *OsMPK3* | CGACTTCGAGCAGAAGGCTCTA | GTTCATCTCGATCGCTTCGTT | Ye et al., 2013 |
| *OsMPK6* | AGGTCACCGCCAAGTACAAG | AGCAGCTTGATCTCCCTGAG | Ye et al., 2013 |

**Reference**

Choi, H. K., Kleinhofs, A., and An, G. (1989). Nucleotide sequence of rice nitrate reductase genes. *Plant. Mol. Biol.* 13, 731-733.

Li, B. Z., Merrick, M., Li, SM., Li, H. Y., Zhu, S. W., Shi, W. M., et al. (2009) .Molecular basis and regulation of ammonium transporter in rice. *Rice. Sci.* 16(4), 314-322.

Lin, C. M., Koh, S., Stacey, G., Yu, S. M., Lin T.Y., and Tsay, Y.F. (2000). Cloning and functional characterization of a constitutively expressed nitrate transporter gene, *OsNRT1*, from rice. *Plant. Physiol*. 122, 379-388.

Li, R., Afsheen, S., Xin, Z. J., Han, X., and Lou, Y. G. (2013). *OsNPR1* negatively regulates herbivore-induced JA and ethylene signaling and plant resistance to a chewing herbivore in rice. *Physiol. Plantarum.* 147(3), 340-351.

Kusano, M., Tabuchi, M., Fukushima, A., Funayama, K., Diaz C., Kobayashi M., et al. (2011). Metabolomics data reveal a crucial role of cytosolic glutamine synthetase 1; 1 in coordinating metabolic balance in rice. *Plant. J*. 66, 456-466.

Ye M., Song Y.Y., Long .J, Wang R.L., Baerson S.R., Pan Z.Q., et al. (2013). Priming of jasmonate-mediated antiherbivore defenseresponses in rice by silicon. *Proc. Natl. Acad. Sci. USA.* 110(38), 3631-3639.
